# Neurobiochemical changes in the vicinity of a nanostructured neural implant

**DOI:** 10.1038/srep35944

**Published:** 2016-10-24

**Authors:** Zsófia Bérces, Kinga Tóth, Gergely Márton, Ildikó Pál, Bálint Kováts-Megyesi, Zoltán Fekete, István Ulbert, Anita Pongrácz

**Affiliations:** 1 Faculty of Information Technology, Pázmány Péter Catholic University, 50/A Práter st., H-1083, Budapest, Hungary; 2 MTA EK NAP B Research Group for Implantable Microsystems, 29-33 Konkoly-Thege st., H-1121, Budapest, Hungary; 3 Institute of Cognitive Neuroscience and Psychology, Research Centre for Natural Sciences, Hungarian Academy of Sciences, 2 Magyar tudósok krt, H-1117, Budapest, Hungary

## Abstract

Neural interface technologies including recording and stimulation electrodes are currently in the early phase of clinical trials aiming to help patients with spinal cord injuries, degenerative disorders, strokes interrupting descending motor pathways, or limb amputations. Their lifetime is of key importance; however, it is limited by the foreign body response of the tissue causing the loss of neurons and a reactive astrogliosis around the implant surface. Improving the biocompatibility of implant surfaces, especially promoting neuronal attachment and regeneration is therefore essential. In our work, bioactive properties of implanted black polySi nanostructured surfaces (520–800 nm long nanopillars with a diameter of 150–200 nm) were investigated and compared to microstructured Si surfaces in eight-week-long *in vivo* experiments. Glial encapsulation and local neuronal cell loss were characterised using GFAP and NeuN immunostaining respectively, followed by systematic image analysis. Regarding the severity of gliosis, no significant difference was observed in the vicinity of the different implant surfaces, however, the number of surviving neurons close to the nanostructured surface was higher than that of the microstructured ones. Our results imply that the functionality of implanted microelectrodes covered by Si nanopillars may lead to improved long-term recordings.

Neural interface technologies are currently being introduced in preclinical applications aiming for the treatment of patients with spinal cord injuries^1^, degenerative disorders^2^, brainstem strokes^3^, amyotrophic lateral sclerosis^4^, tetraplegia^5^ and/or limb amputation(s)^6^. Recording the action potentials of many individual neurons is impossible with non-invasive brain-machine-interfaces (BMI) e.g. EEG, because the neuronal spiking is lost by averaging and filtering across the scalp^1^. The lifetime of invasive, intracortical recording devices such as microfabricated neural probes, however, is limited due to the foreign body response (FBR) of the central nervous system^7^,^8^,^9^. FBR results in the formation of a glial scar, which electrically insulates neurons^10^. On the other hand, reactive astrocytes release proinflammatory and neurotoxic factors that either lead to neuronal death and degeneration, or may inhibit axonal regrowth and regeneration^10^. The immune response around the neural implant can modify the appropriate interpretation of *in vivo* recordings^8^, since it leads to reduced sensitivity, stability and very often to device failure.

Huge efforts are made to overcome the limitation of the chronic use of microelectrodes by improving the biocompatibility of the implant surfaces using bioactive coatings^10^,^11^,^12^,^13^, however, cellular behaviour is influenced both by chemical and physical properties of the environment. Surface topography has been shown to play a role in the biocompatibility of certain materials *in vitro*^14^,^15^,^16^,^17^,^18^,^19^,^20^. Furthermore, it has been demonstrated to clearly modify cellular behaviour: adhesion, migration and differentiation^21^. Since the extracellular matrix (ECM) consists of nanostructured fibrous protein assemblies, the theory that neurons may prefer a similar topography rather than a smooth implant surface seems to be reasonable.

Several groups have investigated the effect of nanostructuring on neurons and glial cells *in vitro* aiming for enhanced *in vivo* neural implant efficiency. Most of them used porous Si^19^,^22^,^23^ or etched Si surfaces with nanometre scale structures^17^,^18^,^24^,^25^,^26^. In other cases, various other materials have been applied, such as GaP^27^ or polymers^28^. In the past few years, by altering the specific surface area, the wetting properties and the pattern regularity of the nanostructured samples, several groups published better neuronal cell adhesion and viability on nanostructured surfaces compared to the smooth references in the past few years^16^,^25^,^26^,^27^.

Early experiments used immortalised neuronal and glial cell lines such as B50 neuronal and LRM55 rat astroglial cells^14^,^15^,^19^,^26^. Later on, primary cells were used as a more realistic model of the *in vivo* effects. Turner and colleagues investigated the attachment of immortalised- and primary astroglial cells on nanostructured surfaces in the same experiment, but ended up with contradictory results^14^. While immortalised astroglial cells preferred to attach to the smoother surfaces, the primary astroglial cells acted the other way around. In the work of Fan *et al.*, primary rat neuronal cells were observed to migrate to nanostructured areas of their samples^16^.

Processes identified in *in vivo* experiments are far more complex than those in widely used *in vitro* model systems. So far, only a few studies can be found in the literature, where the effect of nanostructured surfaces was characterised *in vivo*^8^,^20^,^29^,^30^, and even less where long-term tissue responses were also investigated^8^,^29^.

In our study, bioactive properties of Si nanopillars compared to microstructured Si surfaces were investigated after being implanted for eight weeks, *in vivo*. To form the randomly nanostructured surfaces, we utilise black polycrystalline silicon (poly-Si) thin film. The advantage of black poly-Si is that its production can be integrated into the fabrication scheme of Michigan type silicon neural microelectrodes, since it is made of LPCVD deposited poly-Si thin film and subsequent nanostructuring in a deep reactive ion etching chamber in SF_6_ + O_2_ plasma^31^. The nanoscale morphology of a sensor surface can be tailored by tuning the thickness and morphology of the nanopillar seed layer and the fabrication parameters^32^. The effect of the different surface topographies on glial cells and neurons in the vicinity of the implantation is characterised using GFAP and NeuN immunostaining followed by a systematic image analysis.

## Experimental methods

### Design and fabrication of the microprobes

Our aim was to fabricate test probes with different surface sections for *in vivo* chronic implantation and subsequent histological examination in order to investigate the effect of micro- and nanoscale topography on the surrounding tissue in the same animal. Two types of Si microprobes (referred as reference and nanostructured), with a rectangular cross section of 180 μm × 380 μm and a length of 11 mm were fabricated, with both nanostructured and flat front surfaces. Detailed steps of the fabrication can be found in the Supplementary Material (Supplementary Figure S1).

### Morphological characterization of the different surfaces

Morphologies of the nanostructured surfaces was analyzed using a LEO XB1540 scanning electron microscope (SEM) with 5 keV acceleration voltage and a 30 μm aperture and subsequent image evaluation with ImageJ software. Two quantitative parameters were used to characterise the physical properties of the surface: pillar density and pillar height. Pillar density corresponds to the number of spot-like contaminations on the surface exposed to ion etching^32^,^33^ normalised to 1 μm^2^. Pillar height refers to the average height of BPS nanopillars, and is also determined by the etch rate of the original layer. To identify pillar density, SEM micrographs were acquired at 30° tilt angle. Cross-sectional views at 10° tilt angle were used to measure pillar height. Each parameter was derived from the evaluation of at least three micrographs of the given surface.

The front and backside of the fabricated devices were analysed using a Nanosurf Easyscan 2 atomic force microscope (AFM). Surface roughness was calculated as the ratio of the measured surface area and the projected area using the Gwyddion^34^ image analysing software.

### Implantation procedures

*In vivo* studies were performed on three Wistar rats. In order to reduce the number of animals used, four probes were implanted into each rat, with a total number of 12 implants (6 nanostructured Si, 6 reference probes). Animal care and experiments were carried out in compliance with Animal Care Regulations of the Institute of Cognitive Neuroscience and Psychology of the Hungarian Academy of Sciences and order 243/1988 of the Hungarian Government, which is an adaptation of directive 86/609/EGK of the European Committee Council. The study was approved by the Institutional Animal Care and Use Committee of the Research Centre for Natural Sciences, Hungarian Academy of Sciences. Animals had unlimited access to food and water, when they were awake. Each rat was kept in a 39 cm long, 22 cm wide, 18 cm high cage. Rats were initially anesthetised via intraperitoneal injection of a mixture of 37.5 mg/ml ketamine and 5 mg/ml xylazine at 0.3 ml/100 g body weight. The sleeping state was maintained using intramuscular updates of the same solution (0.3 ml/hour). An electric heating pad was applied for maintaining a body temperature of 37 °C. The anesthetised animals were head-restrained, mounted in a stereotaxic frame (David Kopf Instruments, Tujunga, CA, USA). After shaving the scalp, it was incised in order to gain access to the skull. Soft tissues covering the skull, including the scalp and the periosteum were removed and craniotomy was performed. Four roughly 2 mm × 2 mm windows were drilled for the implants at once so no drilling was necessary post implantation. The stereotaxic coordinates of the four windows in reference to the Bregma were −3 mm/+2 mm in the anterioposterior (AP), −2 mm/+2 mm in the mediolateral (ML) direction (these AP/ML coordinates were thoroughly combined). The openings were kept hydrated using repeated injections of Ringer’s solution onto the exposed surface of the dura mater, which was only incised shortly before each implantation. Implants were organised in such a way that an equal number of nanostructured/reference probes were inserted into the four different craniotomy windows. Nanostructured surfaces faced both medially and laterally to prevent unknown biological effects influencing the results. Positions of the implanted devices is presented in the Supplementary Material on Supplementary Figure S2.

### Immunohistochemistry

Eight weeks after the implantation, rats were perfused under ketamine/xylazine anesthesia (73 mg/kg and 10 mg/kg, respectively), first with physiological saline (5 min) and then with a fixative containing 4% paraformaldehyde and 15% picric acid in 0.1 M phosphate buffer (PB, pH = 7.4, 300 ml). After perfusion the brain was gently taken off from the skull, while the implants were carefully removed from the brain. 60 μm thick horizontal sections were cut from the brains with a Leica 1200S Vibratome. After washing in 0.1 M PB (4 × 10 min), the sections were immersed in 30% sucrose (in 0.1 M PB) for 1–2 days, then freeze-thawed three times over liquid nitrogen, and washed in 0.1 M PB. The endogenous peroxidase activity was blocked using 1% H_2_O_2_ in Tris-buffered saline (TBS, pH = 7.4, 10 min). TBS was used for all the washes (3 × 10 min between each serum) and for dilution of the antisera. The non-specific immunoglobulin binding of the tissue was blocked using 2% normal goat serum (Vector) and 2% normal horse serum (Vector) in TBS (45 min). For the visualization of neurons and glial cells, a monoclonal mouse antibody against neuronal nuclei (NeuN, Millipore, clone A60, 1:2000) and a monoclonal mouse antibody against glial fibrillary acidic protein (GFAP, Millipore, clone GA5, 1:2000) was used for 2 days at 4 °C. For the visualization of immunopositive elements, biotinylated anti-mouse immunoglobulin G (Vector, 1:250) was applied as secondary sera (2 hours) followed by avidin-biotinylated horseradish peroxidase complex (Vector, 1:250, 1.5 hours). After washing the sections in TBS (1 × 10 min) and in Tris buffer (TB, pH = 7.6, 2 × 10 min), they were preincubated for 20 min in 3,3′-diaminobenzidine-tetrahydrochloride hydrate chromogen (DAB, 0.05%, dissolved in TB) and then were developed by 0.01% H_2_O_2_. Brown reaction product accumulated in the immunopositive cells. After washing in TB (2 × 10 min) and PB (2 × 10 min), sections were mounted, dehydrated for light microscopy (2 × 10 min in xilene) and cover-slipped with DePex (Serva).

### Quantitative brain tissue analysis

**Figure 1:**
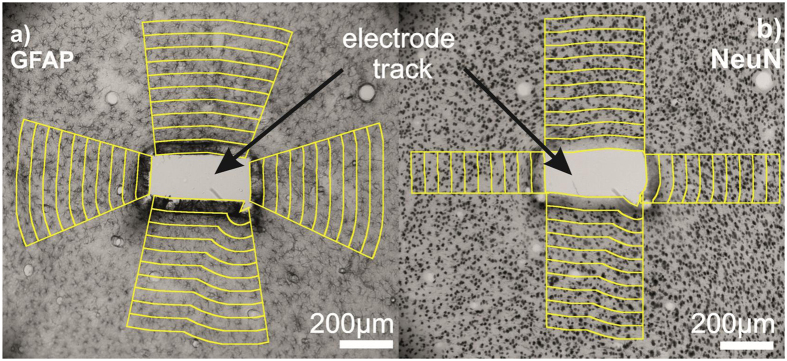
Light micrographs of the electrode tracks were segmented using a custom made ImageJ macro. From the manually defined track outlines 50 μm wide regions were segmented up to 500 μm along the selected shape of the track. Yellow-edge stripes represent the 50 μm wide manually selected ROIs. Different sides of the electrode track were also selected manually. In the case of GFAP staining (**a**), average pixel intensities were calculated in each ROI. On the NeuN stained images (**b**), cell numbers were determined manually in each ROI.

Optical microscopic images were taken of all slices using a Zeiss Axio Scope.A1 microscope and a Jai GO-5000M-PGE digital camera with 2560 × 2048 pixels resolution. Tracks were identified and photographed at 10-fold magnification on each sections using a Zeiss EC Epiplan 10x/0,2 HD objective. The exposure time of each marker was consistently 5 ms. Sections with massive tissue damage and/or fissure around the electrode track were left out from the analysis. Images were segmented using an ImageJ macro into specified regions of interest (ROIs) based on the work of Azemi *et al.*^10^. Electrode track outlines were defined manually. 50 μm wide regions up to the distance of 500 μm were segmented along the selected shape of the track (Fig. 1). The amount of gliosis was determined on GFAP stained sections based on an average pixel intensity calculated in each ROI as a function of the distance from the implantation site. Neuronal cell loss was quantified using NeuN-stained sections. Stained nuclei were counted in each ROI manually, then the average neurons/mm^2^ were estimated as a function of the distance from the track. Measures were normalised to the average pixel intensity or to the cell density of the 400–500 μm zone on the GFAP- or NeuN-stained images respectively. Results were averaged for each distance among the whole image set. Neuron densities show high variability in the different cortical layers. To avoid false results, only ROIs covering the same cortical layer were analysed (Fig. 1b).

### Statistical analysis

Statistical analysis was performed using the IBM SPSS Statistic 22 software. Normality of the data sets was tested using the Shapiro-Wilk test. As the data was found not to be normally distributed, results for the four different surface types were compared at each distance points using Kruskal-Wallis non-parametric test. When a significant difference was found between the groups, Dunn-Bonferroni post-hoc tests were performed. Differences were considered significant in case of p < 0.05. Sample counts were N_Micro-polymer_ = 132, N_MicroSi_  = 66, N_FlatSi_ = 31, N_NanoSi_ =  35 in the case of GFAP and N_Micro-polymer_ = 50, N_MicroSi_ = 25, N_FlatSi_ = 23, N_NanoSi_ = 23 for the NeuN staining.

## Results

### Surface characterization

The morphological parameters of the nanostructured poly-silicon surfaces (***nanoSi***) (pillar density, pillar height) were defined from scanning electron micrographs. The heights of the nanopillars were between 520–800 nm and their density was between 18–70 pillars/μm^2^ depending on the fabrication parameters of the deep reactive ion etching (DRIE) process^32^. The other three surfaces were also visualised using electron microscopy. The microstructured surface has 300–500 nm high surface structures with 500–1000 nm periods and is covered by a fluorocarbon polymer (***micro-polymer***). The fluorocarbon polymer is the reaction product of the cyclic C_4_F_8_ passivation step of the Bosch-process used for deep reactive silicon etching^33^. The microstructured Si surface on the backside of the samples (***microSi***) is in the same size range (1000–2000 nm grain size), but without the deposited fluorocarbon polymer layer. The grain size of the polySi film on the front side of the reference probe is 100–200 nm in diameter, the surface roughness of the front side is 1.04 nm, calculated as the ratio of the measured surface area and the projected area of the AFM images. Therefore this surface is considered to be the flat reference (***flatSi***).

**Figure 2:**
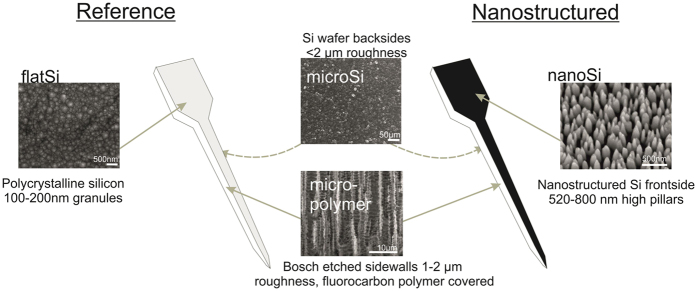
The two types of fabricated devices are one with nanostructured shank (right side) and a reference with polycrystalline Si front-side (left side). The two sidewalls of both devices are microstructured with a fluorocarbon polymer layer on the Si wafer as a result of the Bosch etching step (micro-polymer). The backside of the devices are the non-polished Si wafer (microSi). Front-side of the reference device is a polycrystalline Si layer with 100–150 nm grain size (flatSi), and the front-side of the nanostructured device has 520–800 nm high pillars in a 18–70 pillars/μm^2^ density (nanoSi).

Two types of devices were fabricated with the above described sidewall properties. Figure 2 shows a schematic view of the devices, the representative SEM and AFM images and the properties of each type of surface.

### Reactive astrogliosis

GFAP is an intermediate filament expressed in both immature and mature resting or activated astrocytes^35^. Upon injury or other pathological events in the neural tissue, reactive astrogliosis takes place, including–depending on the severity–proliferation and a cellular hyperthrophy of astrocytes, a glial scar formation and an increased expression of GFAP (for review see ref. 36). Therefore, GFAP staining is a commonly used method to visualise reactive gliosis.

**Figure 3:**
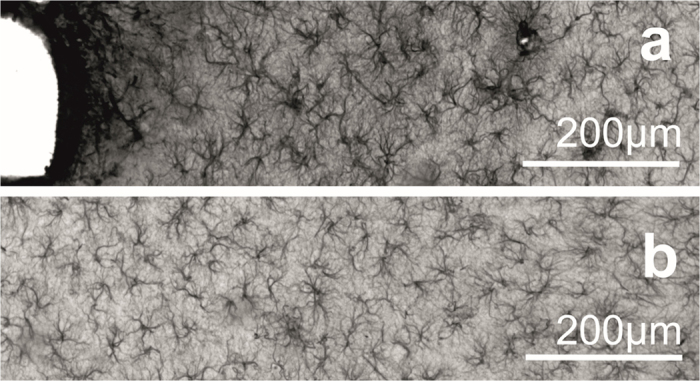
Representative images of GFAP staining. Considerable difference can be seen in the intensity of GFAP staining as a function of the distance (**a**). A massive glial scar is present in the vicinity of the injury. On image (**b**) a part of the stained tissue is presented in a distance over 1 mm from the injury which is considered to be far enough to be intact.

Representative images of GFAP staining are shown in Fig. 3 in the close vicinity of the injury (a) and over 1 mm distance from the implant site (b). Images were taken with the same acquisition parameters. The average pixel intensities of GFAP stained sections were quantified as a function of the distance from the tissue-device interface (Fig. 1a). Quantification procedure was performed in each of the four direction from the interface to visualise the effect of various surface properties. The average intensity curves are plotted as a function of the distance from the implant interface and normalised to the background intensity (400–500 μm).

**Figure 4:**
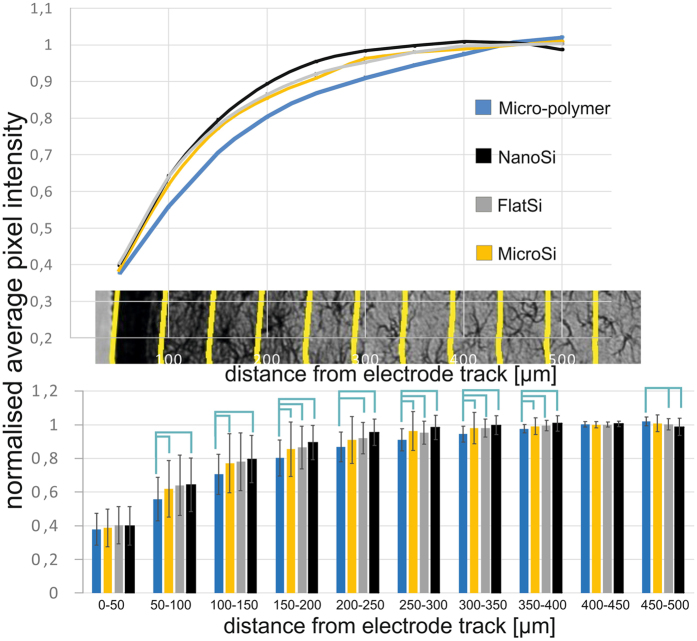
Quantification of the GFAP staining. With increasing distance from the implantation site, the intensity of GFAP staining is decreased. In the vicinity (0–50 μm) of all sides of the electrode track, a massive glial scar was formed regardless of surface properties. From a distance of 50 μm up to 300 μm, the GFAP intensity was consistently lower by 4–5% in case of the nanoSi surfaces than all other types however, differences were not statistically significant. (N_Micro-polymer_ = 132, N_MicroSi_ = 66, N_FlatSi_ = 31, N_NanoSi_ = 35). Sample means and standard deviations are presented. Exact P-values are presented in the Supplementary Material.

As expected, with increasing distance from the implantation site, the intensity of GFAP staining is decreased (Fig. 4). In the vicinity (0–50 μm) of all sides of the electrode track, a massive glial scar was formed regardless of surface properties. From a distance of 50 μm up to 300 μm, the intensity of GFAP staining was consistently lower by 4–5% in the case of the nanostructured surfaces than all other types (N_Micro-polymer_ = 132, N_MicroSi_ = 66, N_FlatSi_ = 31, N_NanoSi_ = 35), however, differences were not statistically significant. Significant differences were shown between the microstructured fluorocarbon polymer covered- (micro-polymer) and the nanostructured Si (nanoSi) surfaces in the distances of 50 μm–500 μm. Between 150 μm–500 μm there were also significant differences between the micro-polymer and the flatSi. Such a difference is more likely to be caused by surface chemistry than topography, since also significant differences were found between microstructured sidewalls (micro-polymer) and unpolished backsides (microSi) in the same distances. FlatSi and nanoSi surfaces do not show significant differences in any distances. Exact P-values for significant differences are presented in the Supplementary Material.

### Neuron density

**Figure 5:**
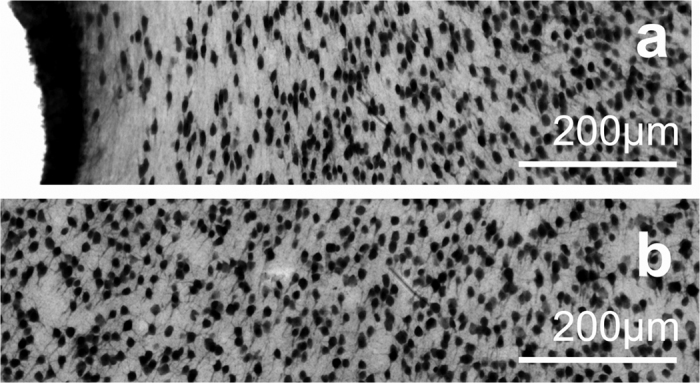
Representative images of NeuN staining. Neuron loss is observed in the vicinity of the injury (**a**). (**b**) Reference cell density at a distance of 1 mm.

The impact of electrode implantation on the surrounding neuronal population was assessed by immunostaining for NeuN, a nuclear antigen found only in neuronal cells. Representative images of NeuN are shown in Fig. 5 in the vicinity of the implant site (a) and in a tissue section over 1 mm from that region (b) considered to be intact. Remarkable neuronal cell loss can be observed in the first 50–100 μm near the implantation track.

The number of neuronal cells on the NeuN-stained sections was counted as a function of the distance from the tissue-device interface at each sides with different surfaces (Fig. 1b). As in the study of Linseimer and colleagues^37^, an automated quantification of neurons resulted in errors and unreliable results due to artefacts and background staining, therefore we applied manual cell counting instead.

The cell count curves are plotted as a function of distance from the tissue-device interface and normalised to the background cell count.

**Figure 6:**
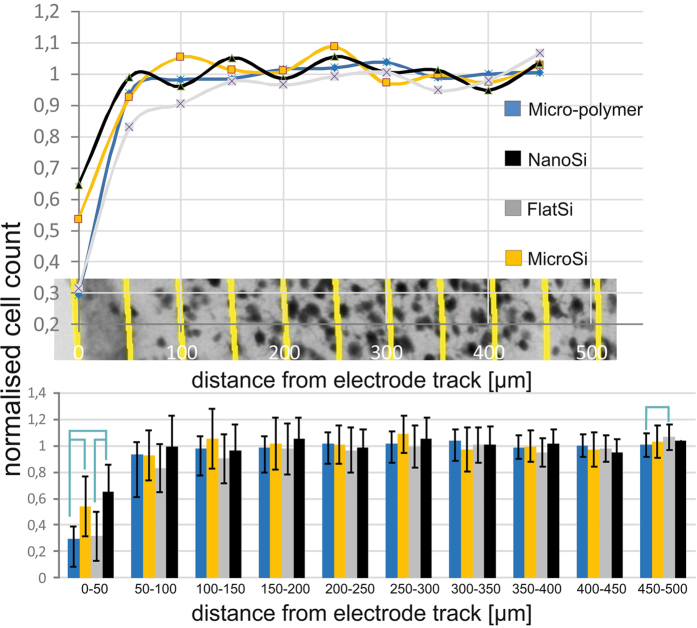
Results of the neural cell count quantification. Increasing neural density can be measured with the distance from the electrode track. After the first 200 μm there was no considerable change in neural cell number in function of the distance. In the first 50 μm, which is considered as the recording distance of a microelectrode, a significant difference was found in average neural cell density in case of surface properties. The highest neural cell loss was found at the microstructured fluorocarbon polymer covered surface, while the highest neural cell density appeared at the proximity of nanostructured implant surfaces. Significant difference was found between the flat and nanostructured Si surfaces implying that nanotopography is favoured by neurons within the distance relevant in neural recording. (N_Micro-polymer_ = 50, N_MicroSi_ = 25, N_FlatSi_ = 23, N_NanoSi_ = 23) Sample means and standard deviations are presented. Exact P-values are presented in the Supplementary Material.

Figure 6 shows increasing neuronal density as the distance from the electrode track increases. After the first 50 μm, there was no remarkable change in neuronal cell number as a function of the distance in case of the examined surfaces. In the first 50 μm, we found a significant difference in neuronal density between the different surfaces examined. The highest neuronal loss was found at the microstructured fluorocarbon polymer covered surface, whereas the highest amount of preserved neurons was seen at the proximity of nanostructured implant surfaces. There was a significant difference between the microSi and micro-polymer surfaces and between nanoSi and micro-polymer surfaces suggesting the relevance of the surface material. Significant difference was also shown between the flat and nanostructured Si surfaces implying that nanotopography is favoured by neurons within the distance relevant in single cell neural recording. Exact P-values for significant differences are presented in the Supplementary Material.

## Discussion

CNS response to chronically implanted microelectrodes leads to recording instability in long-term BMI applications and hinders clinical use. In this study, we investigated the chronic effect of micro- and nanostructured surfaces on the living brain tissue eight weeks after implantation.

This duration is selected, since the foreign body related glial scar formation is supposed to peak 8 weeks after implantation^38^ and recording failure is reported to begin at about 6 weeks^39^.

Recording failure during chronic implantation is a major issue in implantable electrode development. Many groups investigate the effect of different surface modification techniques which are mainly chemical^11^,^13^,^40^,^41^,^42^. *In vitro* experiments performed on nanostructured surfaces provided plenty of promising candidates^16^,^25^,^26^,^27^ however, only a few attempts have been made to verify their biocompatibility as chronically implanted devices in *in vivo* animal studies^8^,^29^,^30^,^43^.

Moxon and colleagues investigated the surface modification on ceramic electrodes with porous Si, *in vivo*^22^. In their experiments, they examined the effect of the contact site surface on electrical signals after one week of implantation time. In their work, they covered the porous Si surfaces with platinum in order to make it conductive and also used a laminin coating on the devices. Due to the filling of the 100 nm size pores with laminin, surface topography is supposed to have a secondary effect only. They eventually observed no difference in the signal-to-noise ratio of the recorded neuronal signal between the covered porous Si and the reference contact site surfaces. Later on, they also investigated the effect of their porous surfaces on the tissue using a histology study^29^. They examined the neuronal cell loss and the development of a glial scar in the case of various surface properties (porous, flat surfaces) using neuronal cell body (NeuN), astroglial intermedier filament (GFAP) and macrophage (ED-1) staining one week after being implanted. They found no difference between the two type of surfaces in the case of glial cells. Nevertheless neuronal cell loss was significantly lower close to the porous surface. On the one hand, it should be noted that the pore sizes were rather in the micron scale in this study. On the other hand, based on literature, it takes at least 3 weeks for glial scar to reach its permanent state^44^, therefore a one-week-long study monitors only the beginning of this process, and not the whole chronic mechanism.

In our study, GFAP staining decreased with the distance from the implant site and the change disappears after around 400 μm. No significant difference could be observed in the GFAP staining in the close vicinity (50 μm) of the device between the different surface properties. A massive uniform glial scar can be seen around all sides of the implanted devices. This result is in agreement with the findings of Moxon and colleagues^29^, where they also observed a difference in the GFAP intensity in the case of structured and flat surfaces, but it was not statistically significant. It should be noted that they studied the tissue after only one week of implantation time, which is not enough for the glial scar to fully develop. However, in the work of Azemi and colleagues, which examined the effect of surface modification of CNS implants with the L1 neuronal adhesion protein, a significant difference was found in GFAP staining between the modified and the reference surfaces after 1, 4 and 8 weeks up to 100 microns from the device-tissue interface^10^. These results suggest that surface topography itself is not sufficient to prevent or reduce gliosis in the closest vicinity of the electrode track.

At larger distance from the implantation site, we found that GFAP staining was consistently lower in the case of the nanostructured Si surfaces compared to the others, although it was significant only in the case of the microstructured fluorocarbon surfaces up to a distance of 350 μm from the track. We found a significant difference between the microstructured Si and fluorocarbon polymer covered surfaces as well. We assume that this effect is due to surface chemistry rather than topography, since the physical dimensions of these two surfaces are in the same size range.

In agreement with the literature, the number of NeuN-positive cells recovered quickly with the increasing distance from the electrode track. No significant cell loss was detected after 50 μm. We found significant differences in the first 50 μm in cell number between the microstructured fluorocarbon polymer and the micro and nanostructured surfaces and also between the reference and nanostructured Si surfaces, which suggests that this effect is due to surface topography. The highest number of preserved neurons was achieved in the case of the nanostructured surface. These differences were observed up to 100 μm, although from 50–100 μm they were not significant any more. This is in agreement with the study of Moxon and colleagues^29^, which reported significantly more neurons up to 100 μm from the track in the case of a porous surface compared to a flat reference one week after implantation. It should be emphasised that in 7 days, only the beginning of the glial scar formation process took place. In the work of Azemi and colleagues, a statistically significant difference was found up to 200 μm in the neuronal cell numbers compared to the reference values (400–500 μm distance) after 1, 4 and 8 weeks^10^. Since L1 protein is reported to support neurite outgrowth, neuronal cell attachment and survival^45^,^46^, they also observed an increased number of neuronal cells close to the L1 covered surface.

Our results and findings are summarised in Table 1.

The proximity of neurons in the first 50 μm of the recording sites is directly related to the functionality and reliability of the implanted device^29^,^37^. In this zone, the reduction of neuronal density has been demonstrated^38^,^47^,^48^,^49^, but it is not clear wether they die, migrate away or are just pushed away by the glial scar, which is formed in the very same zone^37^. Linsmeier and colleagues investigated the correlation between the intensity of GFAP staining and neuronal counts, and found that this correlation was almost non-existent even after 6 weeks.

**Figure 7:**
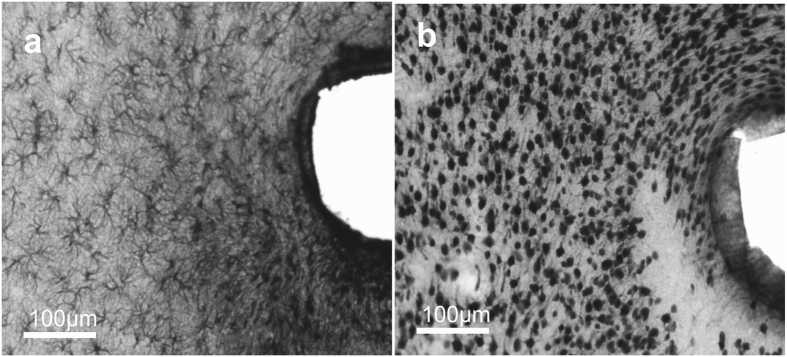
Correlation between the GFAP-positive scar tissue and the neuronal cell loss in the first 100 microns from the implant site. (**a**,**b**) are a pair of adjacent (60 μm distance) tissue slices stained with GFAP (**a**) and NeuN (**b**). Where strong GFAP-positivity is present, indicating the development of the glial scar, neurons are sparse or missing.

In our case, a clear correlation was found between the spatial range of the massive glial scar and the neuronal cell loss in the first 50 μm (Fig. 7).

Beyond this region, neuronal cell densities were similar to the control zone, however the density GFAP-positive fibres, seemed to be increased up to 300 μm.

Based on our findings, nanostructured implant surfaces may improve long-term device functionality more efficiently than flat surfaces. There are more advantages of this passive surface modification over the bioactive surface coating, since nanostructuring poly-Si thin film can be performed during the fabrication of microsystems at a wafer scale. There is no need of individual device treatment, special storage and there is no limited shelf-life. The hypothesis behind these findings has been proposed by several groups in the last few years^30^,^50^,^51^,^52^. Cells in their natural microenvironment interact with the extracellular matrix (ECM) and the surrounding cells by chemical and physical cues. ECM proteins interact with multiple individual cells to coordinate complex multicellular behaviour. These proteins are on a wide size range from several hundred microns (collagen fibers) down to a few tens or a hundred nanometers (collagen fibrins)^53^. ECM structures influence cell shape, polarity and promote migration. It is also known that in several pathological conditions (such as cancer cell invasion) the ECM is remodelled and influences the behaviour of the nearby cells^53^. As the compartments, essential for basic functions of the cells (focal adhesion points, filopodia), are in the size range of these ECM structures, it can be concluded that the interactions between these influence the behaviour of the cells *in vivo*^53^.

During reactive gliosis, a finely graduated continuum of progressive alterations in gene expression and cellular changes occurs to reach an effective defence state of the tissue^36^. Astroglial cells migrate, proliferate and express a special molecular profile. All these mechanisms are influenced by their surroundings: chemical and topological cues of the ECM, the blood components present because of the injury and bleeding, the signals of the injured neurons and also autocrine factors^54^. One can conclude that in such a complex process there should be a pronounced role of the micro- and nano topography of the surroundings besides the chemical cues. Nanostructuring the implant surface in the size range of the physiological ECM structures has a strong potential to assist in reducing the inflammatory process and to prevent it from the secondary effects of the glial response such as secondary neuronal cell death.

Nanostructured surfaces can alter the cell-surface interaction by influencing the composition of the adsorbed protein layer derived from the interstitial fluid and ruptured vasculature of the CNS^55^,^56^. While Denis *et al.*^57^ found that the amount of adsorbed collagen on smooth and nanostructured, functionalised (hydrophobic and hydrophilic) gold thin film is only affected by the surface chemistry, the supramolecular organization of the adsorbed layer is controlled both by surface chemistry and topography. However, Wang *et al.*^58^ systematically characterised bovine serum albumin (BSA) and fibrinogen adsorption on ZnO nanoparticles, nanorods, nanosheets and nanobeams and found that ZnO nanorods compared with other samples had more adsorption sites, which could provide more opportunities for the interaction between material and protein molecules. Nguyen *et al.*^59^ investigated the albumin and fibronectin adsorption on nanostructured black Si surfaces using the combination of atomic force microscopy, Raman spectroscopy, X-ray photoelectron spectroscopy, confocal laser scanning microscopy and scanning electron microscopy. The incremental adsorption of albumin and fibronectin was higher than that measured on the non-structured control surfaces. Adsorption of the two proteins was dissimilar, therefore protein specific.

In our case the specific protein layer composition influenced by the micro and nano topography and also the chemical composition of the surface might play a role in the better survival or regeneration of neurons in the vicinity of nanostructured surfaces. Characterization of CNS specific protein adsorption on the studied nanostructured and microstructured silicon surfaces is beyond the scope of our manuscript, but definitely needs to be carried out in the near future.

Furthermore, nanopatterning the implant surface can have the effect of a diffusion sink for molecules that play a role in the spreading of the inflammatory signal, such as cytokines. Absorbing and immobilising part of these molecules can hinder their role in the reactive gliosis^60^.

It is important to emphasise that many other aspects may affect the short and long term response to implant surfaces. One of the most important ones is likely to be the method and site of implantation. The early steps of gliosis is probably mostly induced by the injury. Some bleeding is inevitable^9^,^61^, but the extent of it depends on the position of the implant and its distance from the larger blood vessels. Another effect to be considered are the micromotions in the brain tissue which is the main reason, why tethered electrodes are reported to cause more tissue damage^37^,^47^.

To evaluate the most local effects of the tissue response, it is necessary to investigate the early stages of gliosis, which is dominated by the response of the microglial cells and factors from the blood in the first 5–7 days^62^. These early released factors stimulate and activate the astroglial cells, thereby have a strong influence on the further formation and severity of the developing glial scar^54^. To visualise the microglial cells and the blood factors present after injury, other specific stainings are needed in a short time (less than 1 week) after implantation.

Furthermore, it is important to investigate the effect of nanostructuring on electrode functionality. In the future, we plan to chronically implant microelectrodes with nanostructured shank and/or contact sites and flat surface references to complete our findings with electrophysiological data. Brain signals will be measured regularly in order to evaluate if there is any effect of surface topography on single unit yield during long time implantation.

## Conclusion

In our study, we investigated the chronic effect of four different surface topographies on the brain tissue *in vivo*. The four different surfaces are the following: (1) polycrystalline Si with 100–200 nm grain size, and with a surface roughness factor of 1.04 (calculated as the ratio of the measured and the projected surface area of AFM images) therefore considered to be the flat reference; (2) the backside of the non-polished Si wafer with a polySi layer with around 2 μm surface patterns; (3) 1–2 μm ridges covered by fluorocarbon polymer; (4) nanostructured surface with a pillar height of 580–800 nm and with a pillar density of 18–70 pillars/μm^2^.

After 8 weeks of implantation time, brain tissue sections were stained with NeuN and GFAP to visualise viable neurons and gliosis, respectively. Optical microscopic images were evaluated using quantitative image analysis. Significantly more neurons were present adjacent to the nanostructured surfaces compared to all the others in the first 50 μm. Less severe gliosis could be observed around the nanostructured surface from 50 μm up to 300 μm compared to the others, but this difference was not significant. A massive glial scar was visualised around all types of the investigated surfaces in the first 50 μm.

Our results suggest that surface topography can alter the effect of implantation regarding the preservation of neurons in a distance of 0–50 μm from the track. Nanostructuring the implant surface may be favourable for long-term applications as a larger neuronal density remains in the vicinity of a nanostructured surface and may provide better signal-to-noise ratio during electric recordings.

Although it is clear that radical changes can be reached by surface adhesive protein coatings, it is still a question how long that solution can work. It will be important to complete these findings with the implantation of functional electrodes and recordings over long time periods to observe the practical effects of surface modifications to the long term stability of CNS implants.

## Additional Information

**How to cite this article**: Bérces, Z. *et al.* Neurobiochemical changes in the vicinity of a nanostructured neural implant. *Sci. Rep.* **6**, 35944; doi: 10.1038/srep35944 (2016).

## Supplementary Material

Supplementary Information

## Figures and Tables

**Table 1:** Summarised results of the quantification of GFAP and NeuN stainings adjacent to our four investigated surfaces (nanoSi, microSi, flatSi, micro-polymer) in three distance intervals (0–50 μm, 50–350 μm, 350–500 μm) noting the significant differences.

	GFAP		NeuN	
0–50 μm	nanoSi	massive glial scar, no difference between surfaces; a clear connection is observed between glial scar formation and neuronal cell loss	nanoSi*^1,2^	highest amount of preserved neurons, significant compared to flatSi and micro-polymer
	microSi		microSi˚	significantly more preserved neurons compared to micro-polymer
	micro-polymer		micro-polymer*^1,^˚	lowest number of preserved neurons, significant compared to microSi and nanoSi
	flatSi		flatSi*^2^	significantly less preserved neurons compared to nanoSi
50–350 μm	nanoSi*	lowest glial intensity, significant difference compared to micro-polymer	nanoSi	no significant cell loss, no difference between surfaces
	microSi	higher glial intensity compared to nanoSi, but not significant	microSi	
	micro-polymer*	highest glial intensity, significant difference compared to nanoSi	micro-polymer	
	flatSi	higher glial intensity compared to nanoSi, but not significant	flatSi	
350–500 μm	nanoSi	no significant difference in staining intensity, can be considered as intact	nanoSi	no significant difference in cell number between surfaces and by distance, can be considered as intact
	microSi		microSi	
	micro-polymer		micro-polymer	
	flatSi		flatSi	
