# Supplementary material for: Neurobiochemical changes in the vicinity of a nanostructured neural implant
Source: Sci Rep. 2016 Oct 24;6:35944. doi: 10.1038/srep35944 (PMC5075914; doi:10.1038/srep35944)
Supplement: Supplementary Information [file srep35944-s1.pdf]

Neurobiochemical changes in the vicinity of a nanostructured neural implant

Zsófia Bérces, Kinga Tóth, Gergely Márton, Ildikó Pál, Bálint Kováts-Megyesi, Zoltán Fekete, István Ulbert, Anita Pongrácz

Supplemetary material

Fabrication

4” (100) oriented, 380 µm thick, single-side polished Si wafers were used as substrates. 500 nm Si-dioxide (SiO_2) was thermally grown to act as an etch-stop layer during later nanostructuring process. 1000 nm thick polycrystalline Si was deposited on top of the SiO_2 by low pressure chemical vapour deposition (LPCVD) in a Tempress oven at 250 sccm flow of SiH_4 gas and at a temperature of 630 °C. Black silicon, made from single crystalline silicon, is a popular bulk material in semiconductor based solar cell industry[1]. It is featured by a nanometre scale pillar-like, rough surface, which provides extremely low reflectivity of the material. Black poly-silicon, first presented by our group[2], offers similar surface quality, but in a thin film material. This unique property provides excellent opportunity to integrate such nanostructured layer in any phase during the thin film processing steps of standard Michigan-type microelectrodes. In our experiment, black poly-Si formation was carried out in an Oxford Plasmalab 100 deep reactive ion etching chamber (DRIE) in mixed mode at cryogenic temperature. First, Microposit S1818 photoresist was used as a masking layer on the reference probes. In order to avoid crack generation in the patterned photoresist, a 40-minute long post-bake at 150°C was performed right after developing of the micropattern. Before dry etching the samples, dummy wafers were used for a preliminary conditioning of the applied recipe for 10 minutes in the cooled (-110 °C) chamber. Relevant etching parameters are summarized in the work of Fekete et al [2]. The used large-scale nanostructuring process is maskless and can be easily integrated in the process flow of a multichannel neural electrode [2], since it relies on a photolithography based MEMS processing of an additional thin film. 300 nm Al film was deposited on both sides of the wafer. The backside Al layer was reinforced with a 4.5 micron thick photoresist coating to provide a mechanically stable etch stop layer for later through-wafer etch. On the front side the layout of the probes was defined by photolithography and a subsequent Al etch. The front side Al film is acting as an etch mask for through-etch of the silicon wafer. The poly-Si/SiO_2 stack was dry etched using CHF_3 and Ar gas mixture. The

through-wafer etch of Si was performed by using the so-called Bosch recipe in a deep reactive ion etching chamber. The Bosch process consists of three basic step. First, SF_6 gas is introduced into the chamber, in our case for 7 seconds. Fluorine radicals and ions, generated in the high density, inductively coupled plasma, attack the exposed silicon surfaces. Ions sputters the sample from a nearly vertical direction. Radicals performs isotropic etch of silicon. After this etching step of silicon, SF_6 is replaced by C_4F_8 for 5 seconds in our case. This step passivates the side-walls with a fluorocarbon layer, which protects vertical surfaces during later cycles. A number of 500-600 etch-deposition cycle was necessary to reach the bottom Al membrane on the wafer. The contour etch is followed by photoresist and Al removal in acetone and in a four component etching solution, respectively. Figure S1 shows a schematic cross-sectional view of the process flow of the nanostructured and reference probe (without black poly-silicon formation) fabrication on the same wafer.

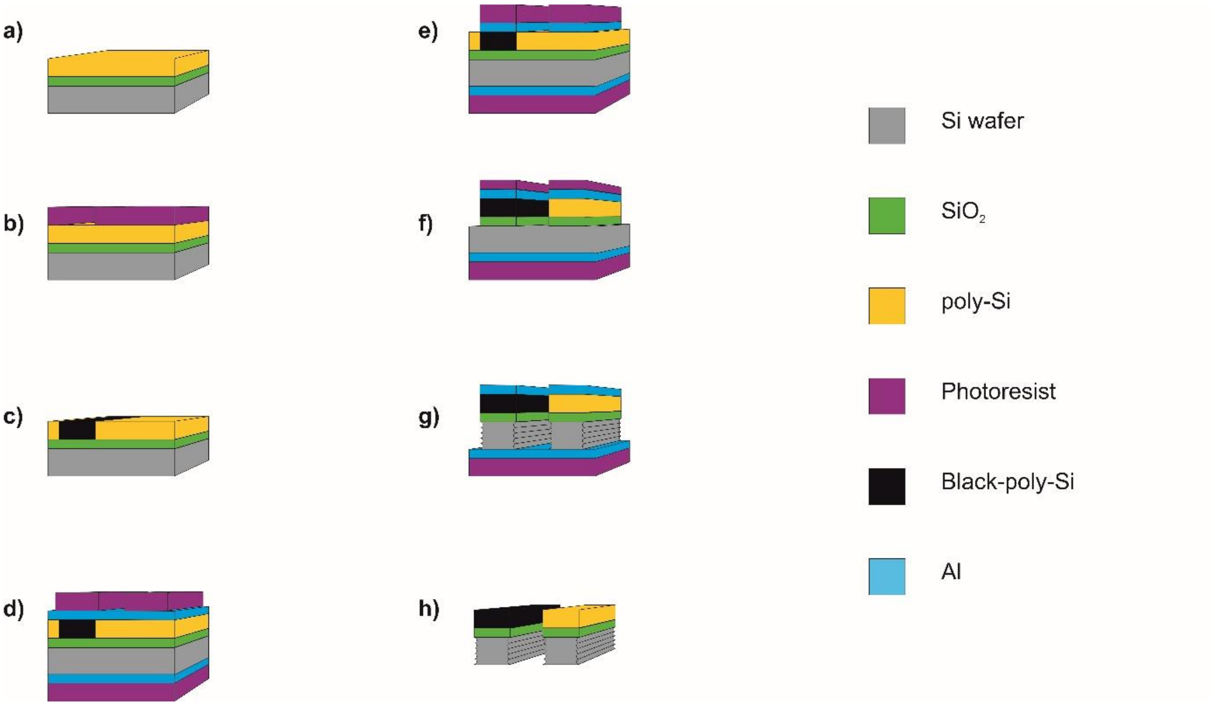

Supplementary Figure S1. Schematic cross sectional view of the microfabrication process of the probes. The fabrication steps are the following: a) thermal SiO_2 growth (500 nm) and polycrystalline Si (1000 nm) deposition, b) photolithography: masking of the nanostructured parts, c) black Si formation by deep reactive ion etching d) deposition of an Al masking layer (300nm) and photoresist for device contour definition e) photolithography, Al etching, f) PolySi and SiO_2 dry etching, g) through-wafer etch of silicon by Bosch recipe, h) photoresist and Al removal from the front- and backside.

Implant positions

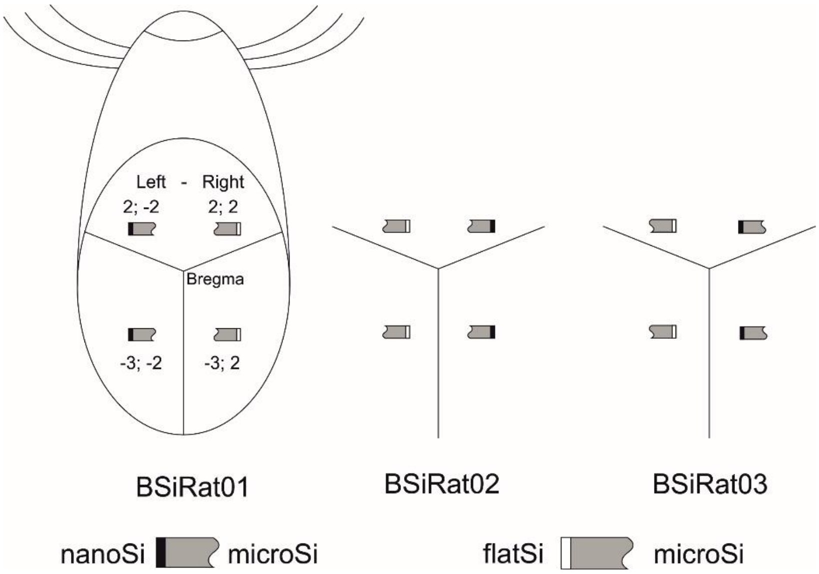

Supplementary Figure S2. Implant positions of the devices. The stereotaxic coordinates of the four windows with respect to the Bregma were -3 mm / +2 mm in the anterioposterior (AP), and -2 mm / +2 mm in the mediolateral (ML) direction. Implants were organized in such a way that an equal number of nanostructured/reference probes were inserted into the four different craniotomy windows. Nanostructured surfaces faced both medially and laterally (Suppl 2.) to prevent unknown biological effects influencing the results.

P-values for statistical tests

GFAP staining

Significant differences were shown between the microstructured fluorocarbon polymer covered- (micro-polymer) and the nanostructured Si (nanoSi) surfaces in the distances of 50 µm – 500 µm.

P-values for micro-polymer and nanoSi:

50-100 µm: 0.032

100-150 µm: 0.008

150-200 µm: <0.001

200-250 µm: <0.001

250-300 µm: <0.001

300-350 µm: <0.001

350-400 µm: <0.001

450-500 µm: <0.001

Between 150 µm-500 µm there were also significant differences between the micro-polymer and the flatSi surfaces.

P-values for micro-polymer and flatSi:

150-200 µm: 0.034

200-250 µm: 0.009

250-300 µm: 0.006

300-350 µm: 0.005

350-400 µm: 0.006

450-500 µm: 0.05

Significant differences were found between microstructured sidewalls (micro-polymer) and unpolished backsides (microSi) in the same distances.

P-values for micro-polymer and microSi:

50-100 µm: 0.037

100-150 µm: 0.022

150-200 µm: 0.048

250-300 µm: <0.001

300-350 µm: 0.006

FlatSi and nanoSi surfaces do not show significant differences in case of any distances.

NeuN staining

There was a significant difference between the microSi and micro-polymer surfaces: P= 0.01) and nanoSi and micro-polymer surfaces P<0.001. Significant difference was also shown between the flat and nanostructured Si surfaces: P<0.001.

References:

1. Savin, H., Repo, P., von Gastrow, G., Ortega, P., Calle, E., et. al., Black silicon solar cells with interdigitated back-contacts achieve 22.1% efficiency. Nature Nanotechnology **10,** 624–628 (2015)

2. Fekete, Z., Horváth, Á. C., Bérces, Z. & Pongrácz, A. Black poly-silicon: A nanostructured seed layer for sensor applications. *Sensors Actuators, A Phys.* **216,** 277–286 (2014).
